# A Randomized Controlled Trial on the Effectiveness of Court-Type Traditional Thai Massage versus Amitriptyline in Patients with Chronic Tension-Type Headache

**DOI:** 10.1155/2015/930175

**Published:** 2015-09-15

**Authors:** Peerada Damapong, Naowarat Kanchanakhan, Wichai Eungpinichpong, Prasobsook Putthapitak, Pongmada Damapong

**Affiliations:** ^1^College of Public Health Sciences, Chulalongkorn University, Bangkok 10330, Thailand; ^2^Research Center in Back, Neck, and Other Joint Pain and Human Performance, Faculty of Associated Medical Sciences, Khon Kaen University, Khon Kaen 40002, Thailand; ^3^Bamnet Narong Hospital, Amphur Bamnet Narong, Chaiyaphum Province 36160, Thailand; ^4^College of Allied Health Science, Suan Sunandha Rajabhat University, Bangkok 10300, Thailand

## Abstract

This study aimed to evaluate the effectiveness of the court-type traditional Thai massage (CTTM) to treat patients with chronic tension-type headaches (CTTHs) comparing with amitriptyline taking. A randomized controlled trial was conducted. Sixty patients diagnosed with CTTH were equally divided into a treatment and a control group. The treatment group received a 45-minute course of CTTM twice per week lasting 4 weeks while the control group was prescribed 25 mg of amitriptyline once a day before bedtime lasting 4 weeks. Outcome measures were evaluated in week 2, week 4 and followed up in week 6 consisting of visual analog scale (VAS), tissue hardness, pressure pain threshold (PPT), and heart rate variability (HRV). The results demonstrated a significant decrease in VAS pain intensity for the CTTM group at different assessment time points while a significant difference occurred in within-group and between-group comparison (*P* < 0.05) for each evaluated measure. Moreover, the tissue hardness of the CTTM group was significantly lower than the control group at week 4 (*P* < 0.05). The PPT and HRV of the CTTM group were significantly increased (*P* < 0.05). CTTM could be an alternative therapy for treatment of patients with CTTHs.

## 1. Introduction

Tension-type headache (TTH) is the most common type of headache that patients always suffer from mild to severe pain that reduces their ability to perform daily activities [1]. TTH can be classified into episodic tension-type headache (ETTH) and chronic tension-type headache (CTTH) [1]. A major difference between these two types of headache is that the former involves pain that lasts no longer than 15 days per month, whereas the latter causes pain that continues at least 15 days per month for over six months [1]. Research shows that ETTH is much more prevalent than CTTH [2]. However, this can vary depending on gender and age. In the United States, for instance, the prevalence of TTH is greater in women than in men (42% versus 36%) [3]. The prevalence of TTH is estimated at 80% to 90% in Thailand [4].

The treatment of TTH is carried out following both pharmacological and nonpharmacological approaches. As for pharmacological approaches, patients are often prescribed acetaminophen, or aspirin, and nonsteroidal anti-inflammatory drugs (NSAIDs) [5]. On the other hand, nonpharmacological approaches involve a wide variety of treatment, such as stress relief techniques, psychotherapy, therapeutic touch, transcutaneous electrical nerve stimulation (TENS), chiropractic, and massage. Nonpharmacological approaches have been found to be superior to pharmacological ones as they contribute to lower risks and undesirable side effects [6].

Among the nonpharmacological approaches, traditional Thai massage (TTM) is an alternative treatment that has been widely practiced to reduce musculoskeletal illnesses in Thailand [7]. TTM can be divided into two types: the popular-type traditional Thai massage and the court-type traditional Thai massage (CTTM). The CTTM, which is the focus of the present study, applies pressure on specific points along the meridian lines using polite gestures since it was used for royal families [8–10]. A reduction of pain intensity resulting from TTM in patients with headache may be explained in terms of physiological effects. Specifically, TTM involves stimulating blood and lymph circulation as well as the sympathetic nervous system through exerting deep and gentle pressure on the skin and muscles. As a result, the flow of nutrients to tissues is enhanced, and the discretion of toxins and residual substances inside the body improves, thereby reducing swelling and pain [11]. Another possible reason lies in the gate control theory. TTM essentially involves the exertion of pressure on the skin and muscles, thereby stimulating pressure receptors and inhibiting the transmission of pain perception at the spinal cord or the “gate” [12–14]. The TTM benefits of pain reduction could be maintained for a few days and some cases up to 15 weeks [15]. Additional research is needed to determine the long term benefits of TTM relative to control conditions [15]. CTTM has been practiced for long time in women at postpartum period to relieve low back pain. Recently, a study verified and supported its effect in this patient population [16]. However, no research has been carried out on the effectiveness of the CTTM in the treatment of CTTH.

A pilot study was carried out on ten subjects who were recruited using the inclusion criteria developed by the International Headache Society (IHS). They received two 45-minute massage sessions over a one-week period. Therapeutic effectiveness was evaluated before and after each treatment. The results revealed that there was a significant reduction in CTTH symptoms after the treatment at *P* < 0.05 (visual analog scale (VAS) before and after the treatment was 6.80 and 2.70, resp.). It was also found that the posttreatment angles of movement in all directions were enhanced at *P* < 0.05 [17]. The results of the study suggested that the CTTM is likely to be an effective treatment for TTH. Therefore, in the present study, a randomized controlled trial was conducted to determine the effectiveness of the CTTM in the treatment of patients with CTTH in comparison with that of a pharmacological approach.

## 2. Materials and Methods

### 2.1. Design

The study design was randomized controlled trial conducted at the Department of Traditional Thai Medicine, Bamnet Narong Hospital, Amphur Bamnet Narong, Chaiyaphum Province, Thailand.

The study was approved by the 1st Ethics Review Committee for Research Involving Human Subjects, Health Science Group, Chulalongkorn University (COA number 052/2557).

### 2.2. Subjects

The patients aged 18–65 years at Bamnet Narong Hospital diagnosed with CTTH according to the criteria of IHS [1] with the inclusion and exclusion criteria below. The sample consisted of 60 patients with CTTH as identified by the score on the VAS of 4 or above [18]. They were randomly assigned to the treatment group or the control group, each with 30 subjects.

The main inclusion criterion was CTTH diagnosed by criteria of IHS [1], for any of the following: headache occurs on ≥15 days per month on average for over six months; headache lasts hours and could also be continuous, and patient suffers from at least two of the following symptoms: (a) bilateral location, (b) pressing/tightening (nonpulsating) quality, (c) mild or moderate intensity, and (d) not being aggravated by routine physical activity such as walking or climbing stairs and experiencing headache without the following symptoms: (a) no more than one of the following: photophobia, phonophobia, or mild nausea; (b) neither moderate nor severe nausea nor vomiting, not being attributed to another disorder, suffering from headaches at least twice a week, experiencing pain with a severity of greater than or equal to 4 on the VAS, being willing to participate, no prior experience with the CTTM, amitriptyline, and other treatments, or prior experience dating back more than 1 week.

Patients were excluded for any of the following: other types of headache not classified as CTTH and history of the following illnesses or disorders: (a) cervical disorders, such as cervical spondylosis, or herniated disc, (b) neurological disorders, such as hemiplegia or paresis, and (c) skin diseases, such as chickenpox or herpes zoster, no communicative ability or inability to follow instructions, and a fever of 38.5°C.

### 2.3. Assessment and Follow-Up Stage

Outcome measures including the VAS, tissue hardness meter and algometer, and the HRV were assessed before the first treatment; assessment was conducted again in weeks 2 and 4 and follow-up in week 6. The assessor was a licensed physical therapist who was blinded on group allocation of the patients.

### 2.4. Measurement Instruments

The researchers used measurement instruments as follows.

The VAS is an instrument for measuring perception of current pain, rated from 0 (no pain) to 10 (most severe pain ever experienced). In this study, the VAS was assessed before the first treatment as well as before and after the treatment at week 2 and week 4 and followed up in week 6. Tissue hardness and pressure pain threshold (PPT) were measured using a tissue hardness meter and algometer (OE-220, ITO/JAPAN). Tissue hardness measurement involved pushing the force sensor of the device on the skin over the trapezius muscle until the beep sound was noted and then stopping pushing and reading the recorded number giving the percentage of tissue hardness. PPT was measured using the algometer mode of the device. The 1-cm^2^ sensor knob was gradually pushed down on the skin over the muscle until the patient feels a little discomfort without pain. At this time, the patient was informed to push the hand-held switch with a beep sound to stop the procedure and read the recorded force for PPT. The reliability of measurement was tested at the beginning of the study and found high for tissue hardness (ICC = 0.97) and PPT (ICC = 0.92). The tissue hardness and the PPT were assessed before the first treatment as well as after the treatment at week 2 and week 4 and followed up in week 6.

Heart rate variability (HRV) refers to the beat-to-beat alterations in heart rate. This can be analysed as either time-domain or frequency-domain. Time-domain analysis is a continuous measurement of intervals of variability of the QRS complex, resulting from the sinus node depolarization of the ventricle, during an electromyography (ECG). The analysis of duration can be exhibited in the forms of mean normal-to-normal (NNI) intervals and the standard deviation of NNI (SDNN). The greater the SDNN, the higher the variability of the heart rates transmitted through the parasympathetic nerve. Frequency-domain analysis generates power spectral density (PSD) results, using precise mathematical calculation to determine the variability of signals in each frequency. The calculation is done nonparametrically and parametrically. The nonparametric analysis is superior in terms of the application of the fast Fourier transformation (FFT). On the other hand, the parametric one produces smoother frequency components, making it easier to distinguish frequency ranges as well as identify a mean frequency number. Additionally, an estimation of the PSD value from a small sample is still precise. Despite its advantages, the parametric analysis is complex and involves confirmation of the suitability of the sample. This HRV is the best indirect method for measuring cardiac autonomic control, including both the sympathetic and the parasympathetic systems [19]. Biocom Heart Rhythm Scanner PE (Biocom Technologies, USA) was used to measure heart rate variability while the patient was sitting on a comfortable chair before the first treatment as well as after the treatment at week 2 and week 4 and followed up in week 6. At the beginning of the study, we found the reliability of HRV was high for both the time-domain HRV (SDDN: ICC = 0.93, RMS-SD: ICC = 0.90, and LF: ICC = 0.93) and frequency-domain (HF: ICC = 0.90, LF/HF: ICC = 0.91).

### 2.5. Intervention

Sixty patients aged 18–65 years who were diagnosed with CTTH according to the criteria of the International Headache Society (IHS) participated. They were randomly allocated into a treatment group and control group. After the preliminary diagnosis, the randomization, and signing of the consent form, the patients were given a 4-week treatment according to the group to which they belonged and a 2-week follow-up. The details are as follows.

**Figure 1:**
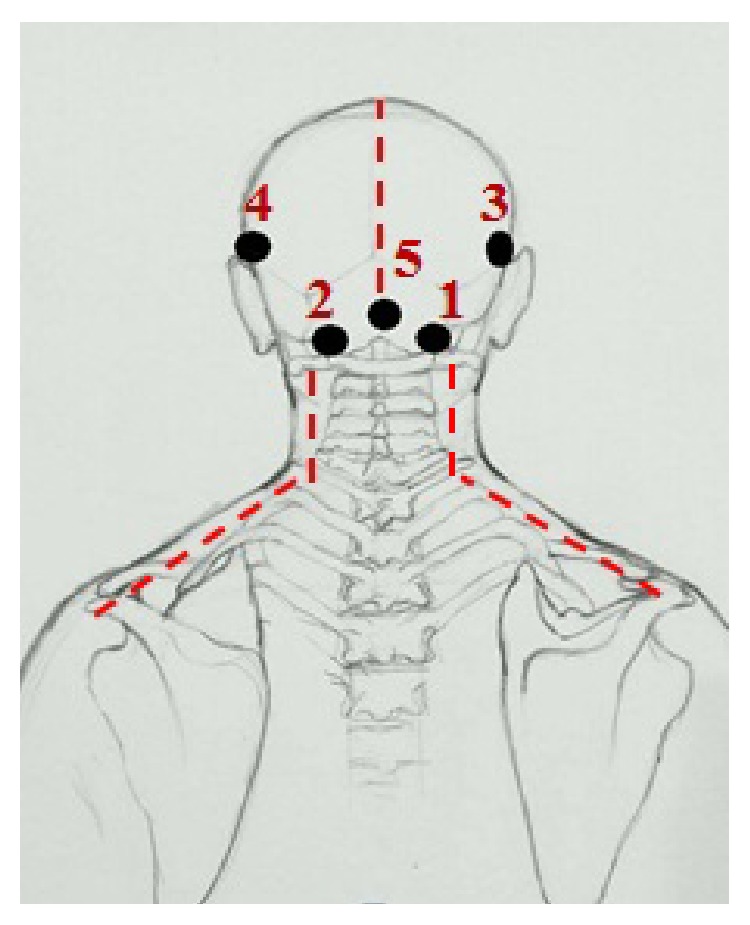
The massage points 1–5 on the back of the head of CTTH patient.

**Figure 2:**
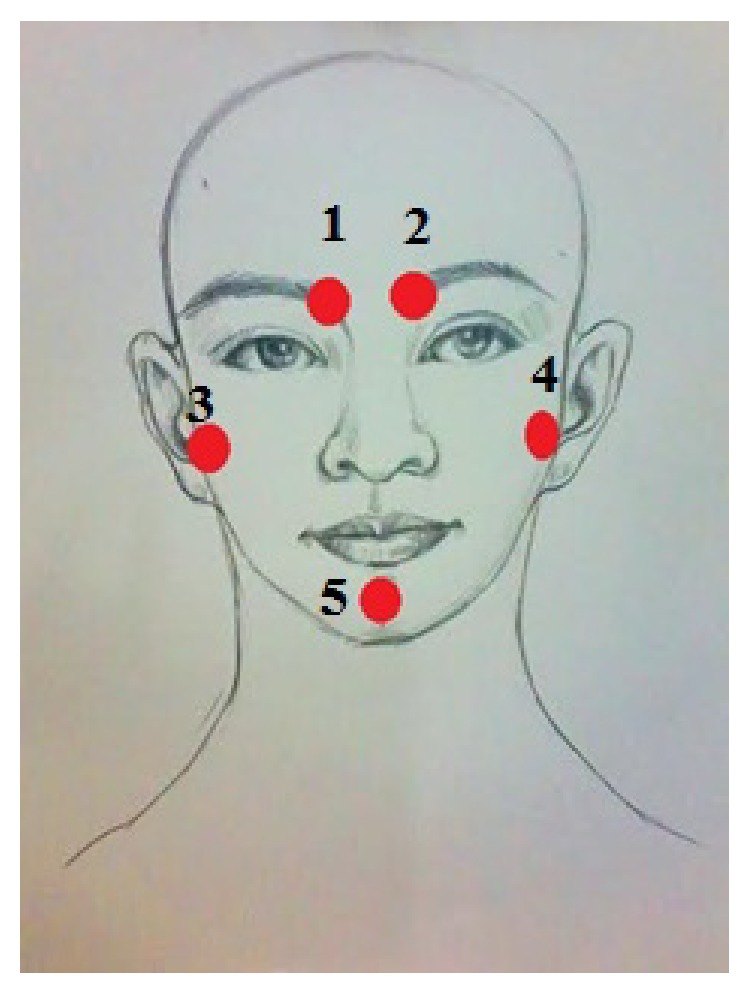
The massage points 1–5 on the forehead of CTTH patient.

The 30 patients who were randomly allocated in the treatment group received the CCTM. The CCTM was done by two licensed applied Thai traditional medical practitioners who have had experience with CCTM more than three years. Lasting 45 minutes for each session, the treatment was conducted twice per week for 4 weeks. A follow-up was done during week 6. The CCTM involved using thumb pressure along the massage meridian lines and points of CCTM (Figures 1 and 2).

In detail, the method for alleviating TTH using CCTM comprised seven steps lasting 45 minutes, starting from the shoulders (15 minutes), both sides of the upper back (5 minutes), the area connecting the neck and the shoulders (10 minutes), the tips of the shoulders (3 minutes), the back of the head (5 minutes) (Figure 1), the middle line of head (2 minutes), and the forehead (5 minutes) (Figure 2).

#### 2.5.1. Shoulder Massage

**Figure 3:**
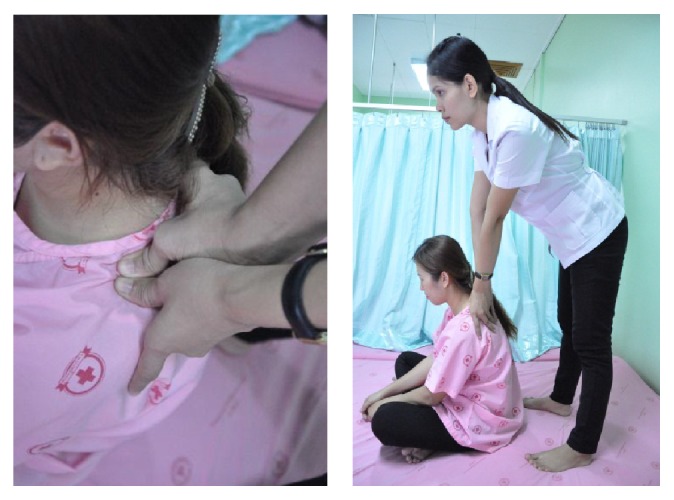
Shoulder massage.

The therapist stood behind the patient, performing one of the following three types of pressure of massage according to the pressure pain threshold of each patient: low-pressure massage, medium-pressure massage, and high-pressure massage. Basically, the thumb pressure must never exceed the pressure threshold of each patient as the therapist has estimated at the beginning of each treatment. The therapist stood with the feet placed slightly apart. For the medium-impact massage, the therapist moved one leg one step behind and bends the other leg slightly. For the high-impact massage, the therapist was in the same posture as for the medium-impact one but increased the bending angle of the trunk and lifted one heel so that she could transfer the body weight more to the thumb pressure. After that, the therapist starts by pressing the thumbs above the shoulder blades, two inches from the medial part of the shoulder tips, and then moving the press along the upper trapezius muscle to the side of C-7 spinous process. In sitting position, pressure from both thumbs is applied started from shoulder to neck and neck to shoulder for two rounds (upper trapezius muscle). Each press lasted 10 seconds (Figure 3).

#### 2.5.2. Back Massage

**Figure 4:**
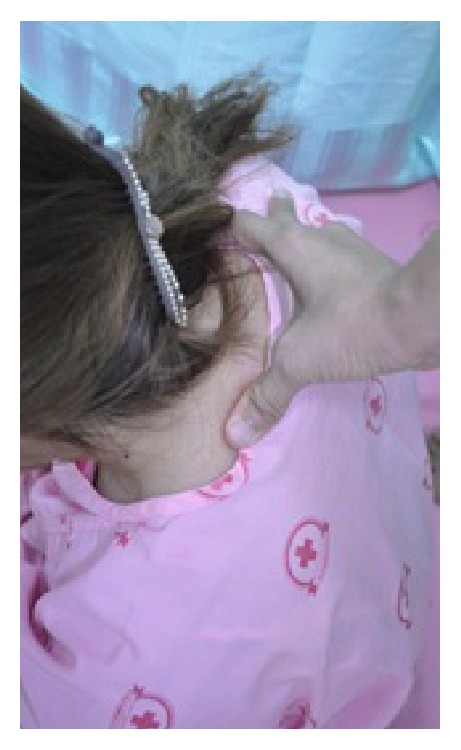
Back massage.

The therapist stood behind the patient, pressing the thumb on the upper trapezius muscle near cervical vertebrae C-7 for 30 seconds (Figure 4).

#### 2.5.3. Basic Massage of the Neck

**Figure 5:**
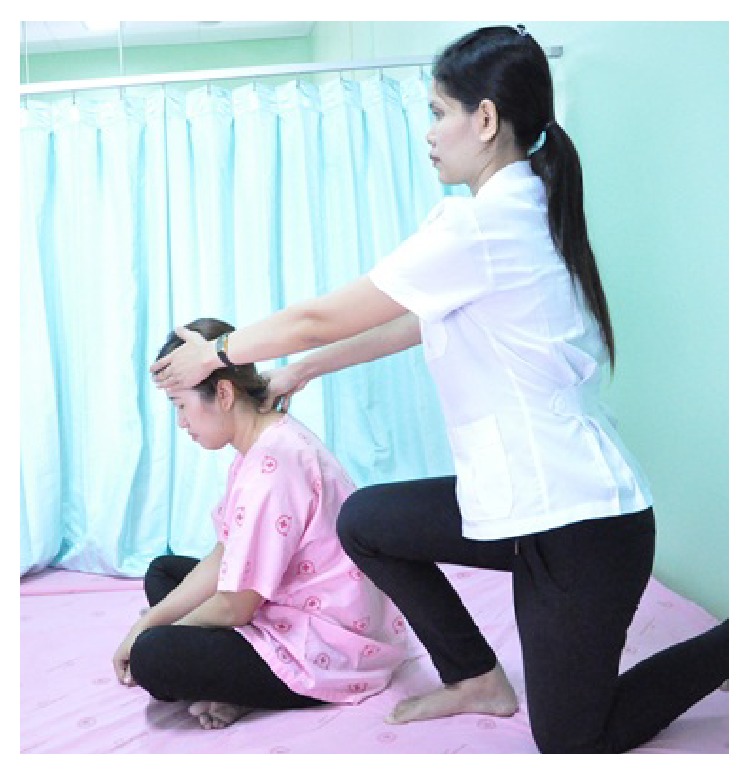
Basic massage of the neck.

The therapist sat with his knees on the floor behind the patient; pressure from thumb was applied at side of the neck while the other hand of the therapist touched (as counter force) the subject's forehead. Neck massage was started from C-7 (upper trapezius and splenius muscle) to the occipital area. After that, right side of the neck was also massaged with each press for 10 seconds (Figure 5).

#### 2.5.4. Shoulder Tip Massage

**Figure 6:**
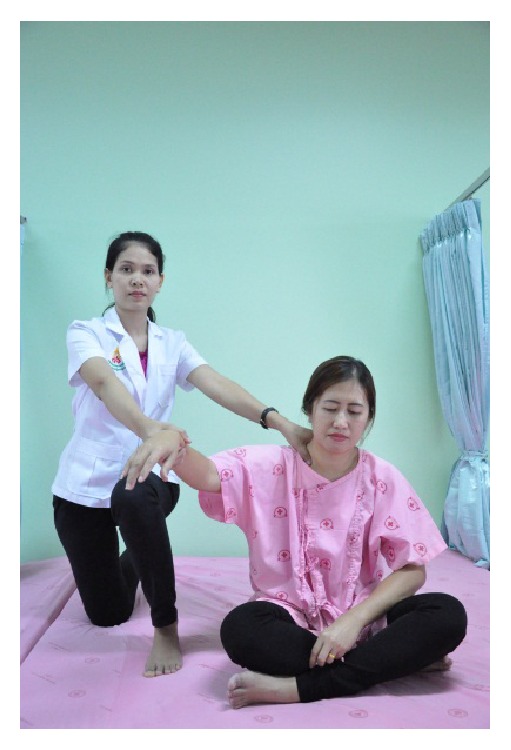
Shoulder tip massage.

The therapist sat with his knees on the floor beside the patient, holding the wrist of the patient with the hand on the same side of the patient's shoulder. The therapist applied thumb pressure using the thumb of the other hand to press on the trapezius and supraspinatus muscles on the suprascapular fossa and maintained each press for 30 seconds (Figure 6).

#### 2.5.5. Massage on the Back of the Head

**Figure 7:**
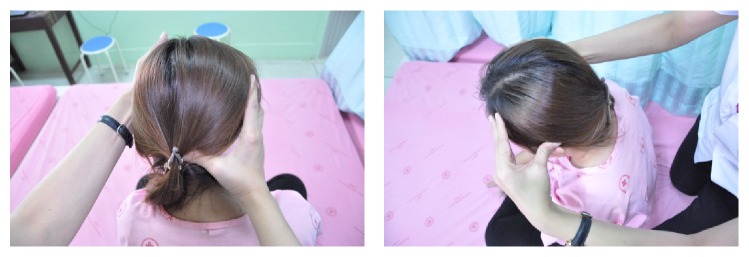
Massage on the back of the head.

The therapist sits with his knees on the floor behind the patient, pressing the thumbs on signals 1–5 of the back of the head and maintaining each press for 30 seconds (Figures 1 and 7).

#### 2.5.6. Massage on the Middle of the Head

**Figure 8:**
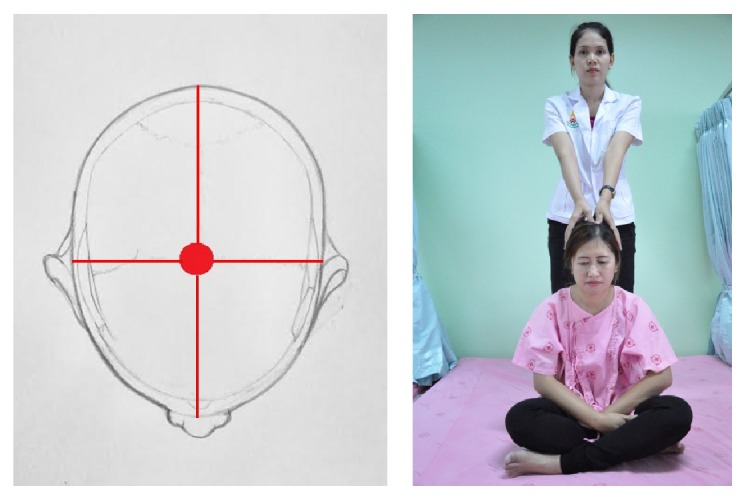
Massage on the middle of the head.

The therapist stood behind the patient and pressed on the skull using the thumbs along midline of the head. The therapist held each press for 30 seconds (Figure 8).

#### 2.5.7. Face Massage

**Figure 9:**
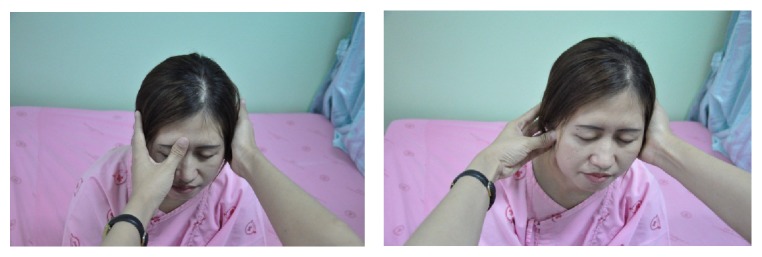
Face massage.

The therapist sat with his knees on the floor in front of the patient, using one thumb to press on each of the five points on the face, and maintained each press for 30 seconds (Figures 2 and 9).

To minimize bruises, the intensity of a massage was adjusted to suit each individual patient's pressure pain threshold and age, drawing on information from therapist observation and inquiries made to the patient regarding his/her feelings. In addition, the patients were requested to inform the therapist immediately if they experienced pain caused by excessive massage intensity. During each CTTH therapy session, the subjects suffering from bruises were treated with topical herbal press. If bruises broke out later, the participants could telephone those in the research team anytime.

The other 30 patients who were randomly allocated into the control group were given amitriptyline by a licensed medical practitioner. They were prescribed 25 mg once daily before bedtime for 4 weeks, and a follow-up was carried out in week 6. Each of them was informed that the medication could cause drowsiness and recommended that strict adherence to the prescribed time of consumption was required.

### 2.6. Randomization

The patients meeting the inclusion criteria were assigned to either the treatment group (receiving CTTM) or the control group (taking amitriptyline) using the simple random sampling technique. The randomization was performed using a lottery by the researcher assistant.

### 2.7. Statistical Analysis

The data was analyzed in terms of mean and standard deviation (SD) for continuous variables and percentage for categorical variables. The study aimed to analyze each session of treatment separately at different time points: before the first treatment, after week 2, after week 4, and after week 6 (during the follow-up). All the analysis was performed on the basis of intention to treat.

An analysis of Repeated Measures ANOVA was used to compare the means of within-group data, and analysis of covariance (ANCOVA) was also conducted to compare the differences between the two groups as well as estimate the adjusted difference between the two groups at 95% confidence level. Post hoc tests using Fisher's Least Significant Difference (LSD) were applied for multiple comparisons.

## 3. Results

**Figure 10:**
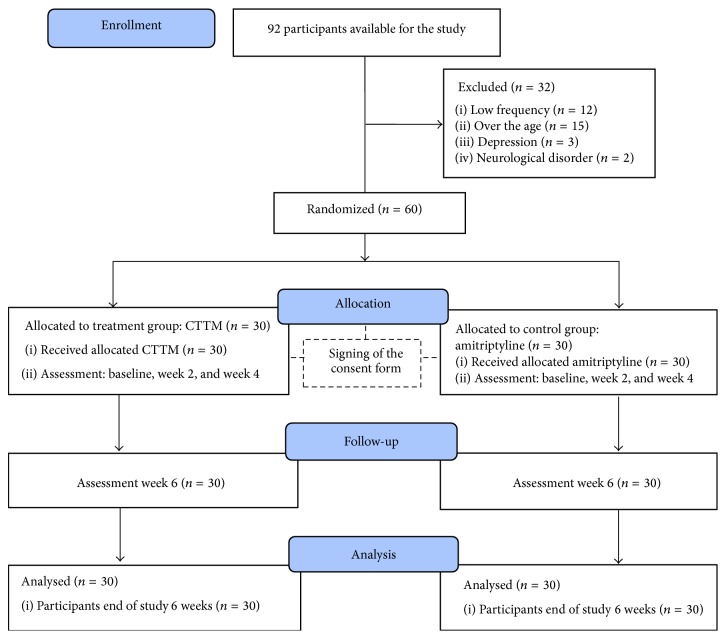
Flow chart of entry and discontinuation by participants during the study.

A total of ninety-two subjects responded to the recruitment advertisements and were screened for eligibility for the study. After screening by medical doctor, thirty-two subjects dropped out because of low frequency (*n* = 12), being over the age (*n* = 15), depression (*n* = 3), and neurological disorder (*n* = 2); sixty subjects met the inclusion criteria and signed the consent forms. Thirty subjects were randomly selected to receive CTTM and the others were the control group. At 6 weeks of the follow-up phase, a detailed summary of patient recruitment, participation, attribution, and reasons for exclusion from the study is presented in Figure 10.

Details of demographic data were presented in Table 1. There were no significant differences between the groups.  Table 2 summarized the results for patient-rated outcome repeated measures at all assessment time points during the baseline, week 2, and week 4 of treatment and at week 6 follow-up after final treatment. The outcomes of VAS, tissue hardness, and PPT were compared within group at week 2, week 4, and week 6 follow-up.

The results showed a statistically significant increase in the outcome means for both the groups (*P* < 0.05).  Table 3 presented comparison of the adjusted mean and 95% CI of outcome measures at each assessment time point. It was found that, after adjustment for baseline levels, the VAS, tissue hardness means were statistically different at week 4 (*P* < 0.05) and PPT means were statistically different at week 2 (*P* < 0.05), week 4 (*P* < 0.05), and week 6 follow-up (*P* < 0.05). In addition, a greater fall in tissue hardness was found for the CTTM group than for the control group.

**Figure 11:**
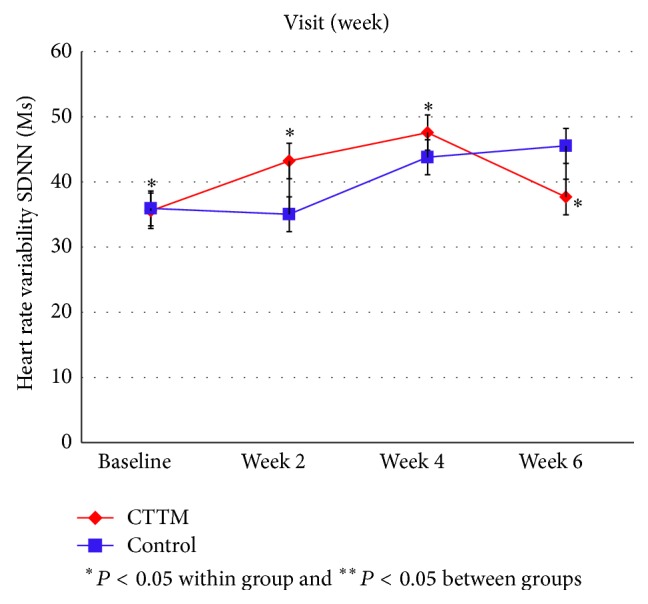
Heart rate variability by SDNN.

**Figure 12 fig12:**
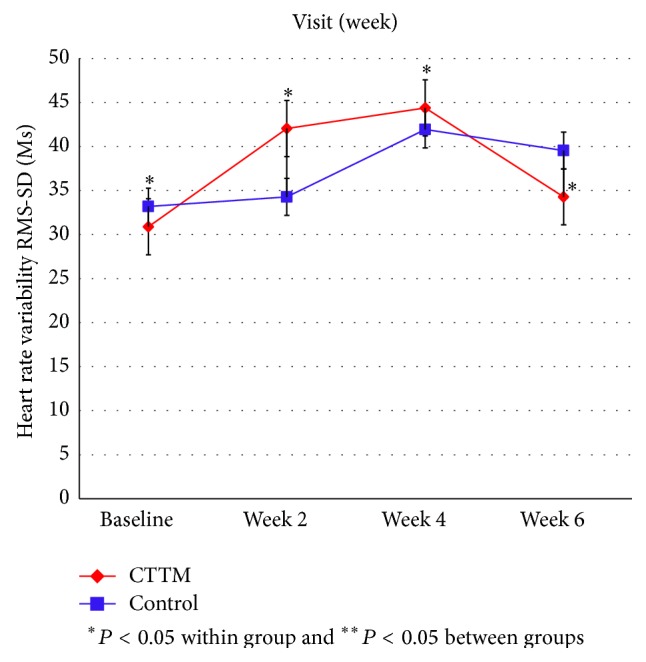
Heart rate variability by RMS-SD.

**Figure 13 fig13:**
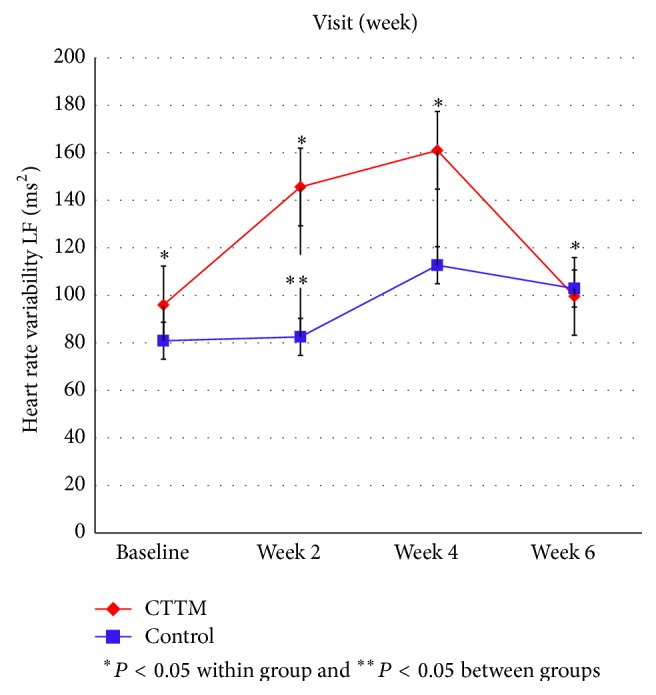
Heart rate variability by LF.

**Figure 14 fig14:**
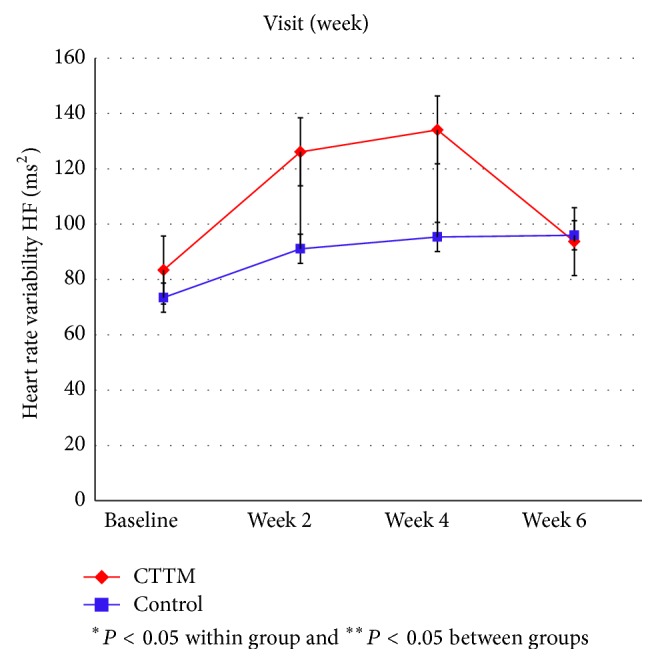
Heart rate variability by HF.

**Figure 15:**
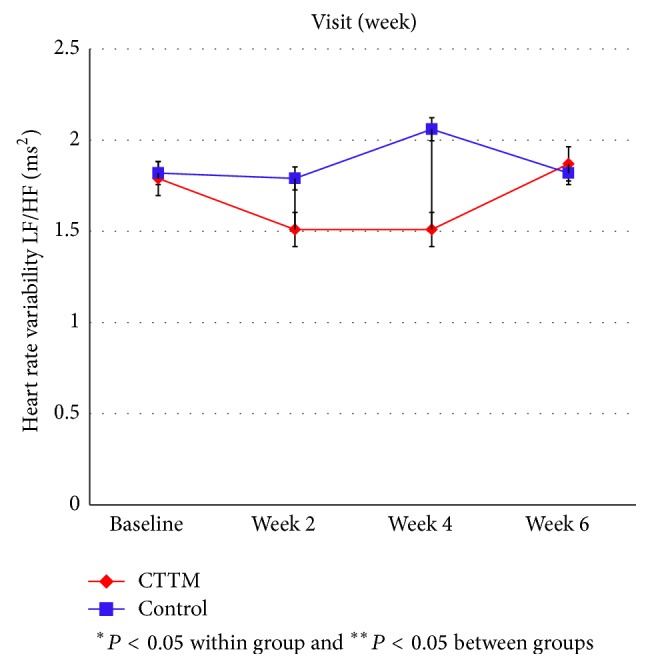
Heart rate variability by LF/HF.

Figures 11 [Fig fig12] [Fig fig13] [Fig fig14]–15 showed the comparison of the within-group means for the HRV of the CTTM group and the control group through time-domain analysis of SDNN and RMS-SD as well as a frequency-domain analysis of LF, HF, and LF/HF at different assessment time points. We found that the values of SDNN, RMS-SD, and HF for the CTTM group increased significantly (*P* < 0.05). However, the control group also exhibited similar results. When the CTTM group and the control group were compared, it was found that the LF value was statistically different at week 2 (*P* < 0.05)

## 4. Discussion

This study aimed to determine the effectiveness of the court-type traditional Thai massage (CTTM) in treating patients suffering from chronic tension-type headaches (CTTHs) in comparison with amitriptyline. Assessment was conducted at week 2, week 4, and week 6 follow-up for both the CTTM group and the control group. The outcome measures involved visual analog scale (VAS), tissue hardness, pressure pain threshold (PPT), and heart rate variability (HRV).

The headache pain intensity scores reduced from baseline at week 2, week 4, and week 6 follow-up for both the CTTM group and the control group. In terms of the VAS, a comparison between the two groups indicated statistically significant differences for all assessment time points. Such a decline in headache pain intensity may be explained in terms of physiological effects. Specifically, TTM involves stimulating blood and lymph circulation as well as the sympathetic nervous system through exerting pressure on the skin and muscles. As a result, the flow of nutrients to tissues is enhanced, and the discretion of toxins and residual substances inside the body improves, thereby reducing swelling and pain [11]. Another possible reason lies in the gate control theory. TTM essentially involves the exertion of pressure on the skin and muscles, thereby stimulating pressure receptors and inhibiting the transmission of pain receptors at the spinal cord or the “gate” [12–14]. Finally, TTM may relieve muscle tension by freeing the mind from stress and anxiety.

The results were consistent with Chatchawan and colleagues [20] on the effects of Thai traditional massage on pressure pain threshold and headache intensity in patients with chronic tension-type and migraine headaches. Seventy-two participants who had had a headache diagnosis for at least 3 months before the experiment were recruited and randomly allocated in either a massage group or a control group (medication). After the treatment, and at 3 and 9 weeks of follow-up, in both groups, headache intensity decreased significantly (*P* < 0.05). However, they found no significant differences between the groups (*P* > 0.05). Similar findings were also reported elsewhere. Kanji and colleagues [21] determined efficacy of regular sauna bathing for chronic tension-type headache and found headache intensity significantly differed between the sauna and control group by 1.27 (*P* = 0.002).

A comparison of the effectiveness in reducing tissue hardness within-group means for tissue hardness of the CTTM group and the control group at baseline, week 2, week 4, and week 6 follow-up indicated a significant decline for both the groups (*P* = 0.001).

When the CTTM group and the control group were compared at each assessment time point, it was found that the two groups differed significantly at week 4 (*P* < 0.05) with the tissue hardness value for the former being lower than that of the latter. In addition, tissue hardness generally reduced for both the groups at all assessment time points. All this seems to point to the effectiveness of CTTM in improving tissue hardness and the superiority of CTTM over amitriptyline when a series of massage treatment is administered.

Similar findings are also reported elsewhere. Zheng et al. [22] evaluated the therapeutic effectiveness of lumbar tender point deep massage in treating chronic nonspecific low back pain. They found that the increase in muscle hardness after the treatment was statistically significant (*P* < 0.05) [23].

A comparison of the effectiveness in increasing pressure pain threshold within-group means of the CTTM group and the control group assessed at baseline, week 2, week 4, and week 6 follow-up indicated a significant increase in PPT at all assessment time points for both the groups (*P* = 0.001). When the CTTM group and the control group were compared, it was found that the two groups differed significantly at all assessment time points (*P* < 0.05) with the mean difference equaling 0.28, 0.35, 0.32, and 0.38, respectively. However, the figures did not exceed 1 (clinical significance set at 1 kg/cm^2^ or 2.2 lb/cm^2^) [24], demonstrating no clinically significant difference.

The results are consistent with those of other researches carried out earlier. Kruapanich and colleagues [18] compared the effectiveness of TTM and taking a nap in treating ETTH patients. The study revealed that TTM could increase PPT by as much as 0.41 kg/cm^2^ (or 0.90 lb/cm^2^) and that the difference between the two groups was statistically significant (*P* < 0.001) but not clinically significant as the degree of difference was lower than 1. Sooktho and colleagues [25] examined the therapeutic effectiveness of TTM in treating CTTH and migraine patients, indicating improvement at week 3 and week 6 follow-up but no clinically significant difference between the TTM group and the control group. Similar findings are also reported, Chatchawan and colleagues [20], on effects of Thai traditional massage on pressure pain threshold and headache intensity in patients with chronic tension-type and migraine headaches. After the treatment and at 3 and 9 weeks of follow-up, the TTM group showed a significant increase in PPT (*P* < 0.01) compared with the sham ultrasound group.

On the other hand, the present findings do not resemble those reported in Toro-Velasco et al. [26], which investigated the effectiveness of a head-neck massage protocol in alleviating CTTHs compared to placebo ultrasound. An assessment of PPT at both sides of temporalis muscles immediately and 24 hours after the treatment did not demonstrate an improvement in the patients' conditions. It should be noted, however, that Toro-Velasco et al.'s research is different from the present study in terms of research design, sample size, and the form and area of massage.

The improvement in PPT reported in this study may be explained as follows. In CTTH patients, trigger points (TrPs) may be found around the head, temporal, occipital bone, shoulders, and eyes [27–30]. As the present research involves administering massage around these areas according to the CTTM, PPT is likely to increase with a decline in pain sensitivity. This explanation is supported by the findings of Simons [31], which reported that massage and muscle stretching could relieve muscle tension and hence muscle pain sensitivity.

A comparison of the effectiveness in increasing heart rate variability within-group means of the CTTM group and the control group through a time-domain analysis of SDNN and RMS-SD as well as a frequency-domain analysis of LF, HF, and LF/HF at different assessment time points showed that the values of SDNN, RMS-SD, and HF for the CTTM group increased significantly (*P* < 0.05). These results indicate reduced stress characterized by increased HRV through the parasympathetic nervous system.

When the CTTM group and the control group were compared, it was found that the LF value was statistically different at week 2 (*P* < 0.05) and the HF value was statistically different at day 1 (*P* < 0.05). Although no statistically significant difference was identified between the two groups, the values of LF, HF, and LF/HF all revealed increased HRV for both groups. These findings were similar to those reported in Toro-Velasco et al. [26], which found that a head-neck massage protocol was effective in increasing HRV and that the difference between the treatment group and the control group was statistically significant. In addition, Buttagat et al. [32] examined the immediate effects of TTM on improving HRV and stress-related parameters in patients experiencing back pain with myofascial TrPs, reporting results consistent with those in the present study. Specifically, it was found that TTM could increase HRV, which characterizes an improvement in the function of the parasympathetic nervous system (*P* < 0.01) and that such an improvement was not significant for the control group.

All the findings suggest that CTTM tends to be an effective therapy that can enhance the function of the parasympathetic nervous system, thereby reducing tension in CTTH patients.

Regarding side effects, we found some of the patients who had never received massage therapy before reported experiencing mild ache in the shoulders after the first treatment that subsided within one to two days. As for the control group, some of the patients reported morning drowsiness during the first few days of consumption of amitriptyline, so they were advised to have enough rest.

## 5. Conclusions

This research compares the effectiveness of CTTM and amitriptyline in treating CTTH patients. In terms of VAS the results showed a statistically significant decrease in headache pain intensity for the CTTM group at different assessment time points and a statistically significant difference between the CTTM group and the control group at each assessment time point. The superiority of CTTM over amitriptyline was also identified for other variables. As for tissue hardness, the value for the CTTM group was significantly lower than that of the control group at week 4, and the value for both the groups reduced at the other assessment time points, although not statistically significant. Additionally, the PPT of the CTTM group increased significantly and was significantly higher than that of the control group. Finally, the HRV of the CTTM group increased significantly in terms of SDNN, RMS-SD, and LF. It can therefore be concluded from the findings that CTTM seems to be an effective therapy for enhancing the function of the parasympathetic nervous system and other stress-related variables as well as reducing CTTHs.

## Limitations of the Study

Double blinded assessment was not applicable in this study since each of the patients knew the group they belonged to. However, blinded assessor was the only thing we could do apart from random allocation for compromising the internal validity of the study.

## Recommendations for Further Study

Based on the present findings, the CTTM could serve as an alternative therapy for the treatment of CTTH patients. This could be an appropriate therapy for the patients who suffer with adverse effects of medication. Further research should determine long term effects of these two types of treatment because some patients in the study were fully recovered within one month of treatment and two weeks of follow-up whereas the others were not. Effects of longer period of treatment and longer follow-up period are not known.

## Figures and Tables

**Table 1:** Demographic data.

Characteristics	CTTM	Control	*P* value
	*n* = 30 (%)	*n* = 30 (%)	
Gender			
Female	26 (86.70)	29 (96.70)	<0.05
Male	4 (13.30)	1 (3.30)	
Aged (year)			
23–36 years	6 (20.00)	1 (3.30)	0.350
37–50 years	13 (43.30)	13 (43.30)	
51–64 years	11 (36.70)	16 (53.30)	
*Mean = 49.75; SD = 10.93; median= 48 *			
*Classify from class interval *			
Occupation			
Agriculture	9 (30.00)	10 (33.30)	0.939
Self-employed/business	4 (13.30)	3 (10.00)	
Government officer/government employer	8 (26.70)	7 (23.30)	
Work as employee	9 (30.00)	10 (33.30)	
Underlying diseases			
None	25 (83.30)	26 (86.70)	0.690
Yes (allergy)	2 (6.70)	1 (3.30)	
Yes (diabetes mellitus)	1 (3.30)	2 (6.70)	
Yes (hypertension)	2 (6.70)	1 (3.30)	
History of headache in the lifetime (time)			
Headache frequency of life (time) ≥ 50 times	30 (100.00)	30 (100.00)	1.000
Duration time in each headache attack			
<30 minutes	1 (3.30)	1 (3.30)	1.000
30 minutes to 1 hour	18 (60.00)	18 (60.00)	
1 hour to 2 hours	6 (20.00)	6 (20.00)	
> a day <7 days	5 (16.70)	5 (16.70)	
Working affected by headache			
None	1 (3.30)	4 (13.30)	0.926
Can work but less than normal	18 (60.00)	16 (53.30)	
Can work if it is necessary	6 (20.00)	6 (20.00)	
Cannot work (stop working)	5 (16.70)	4 (13.30)	
Previous treatments of headache			
Rest	9 (30.00)	5 (16.70)	0.935
Drug	11 (36.70)	12 (40.00)	
Medical doctor	10 (33.30)	13 (43.30)	
Baseline of clinical outcome measure			
Visual analog scale (VAS 0–10 cm); mean ± SD	6.30 ± 1.20	6.06 ± 0.94	0.105
Tissue hardness (%); mean ± SD	59.89 ± 11.04	57.16 ± 8.50	0.159
Pressure pain threshold (kg/cm^2^); mean ± SD	3.17 ± 0.69	2.85 ± 0.79	0.264
Heart rate variability (HRV); mean ± SD			
Standard deviation from the mean RR value; SDNN (Ms)	35.57 ± 13.38	35.93 ± 24.46	0.119
Root mean square of the standard deviation; RMS-SD (Ms)	30.89 ± 15.40	33.18 ± 30.01	0.162
Low frequency power; LF (ms^2^)	95.97 ± 72.94	80.87 ± 76.01	0.724
High frequency power; HF (ms^2^)	83.43 ± 75.74	73.47 ± 74.60	0.654
Low frequency to high frequency ratio; LF/HF (ms^2^)	1.79 ± 1.52	1.82 ± 1.51	0.933

**Table 2:** Patient-rated outcome repeated measures at all assessment time points during the baseline, week 2, and week 4 of treatment and at week 6 follow-up after final treatment (Repeated Measures ANOVA).

Outcome	Group	Baseline	2-week follow-up	4-week follow-up	6-week follow-up	*P* value
			(Mean ± SD)	(Mean ± SD)	(Mean ± SD)	
Visual analog scale (VAS 0–10 cm)	CTTM	6.3 ± 1.20	3.73 ± 1.22	2.90 ± 0.95	2.60 ± 0.72	<0.05
	Control	6.0 ± 0.94	4.40 ± 1.37	3.50 ± 1.27	2.90 ± 1.06	<0.05

Tissue hardness (%)	CTTM	59.89 ± 11.04	48.85 ± 11.29	46.20 ± 7.54	48.96 ± 8.01	<0.05
	Control	57.16 ± 8.50	49.80 ± 10.45	49.51 ± 7.85	47.41 ± 8.62	<0.05

Pressure pain threshold (kg/cm^2^)	CTTM	3.17 ± 0.69	3.72 ± 0.60	4.01 ± 0.62	4.12 ± 0.55	<0.05
	Control	2.85 ± 0.79	3.17 ± 0.65	3.48 ± 0.68	3.53 ± 0.73	<0.05

*Note*. CTTM is court-type traditional Thai massage. NA is not available. *P* < 0.05 is statistically significant differences from baseline.

**Table 3:** Comparison of the adjusted mean and 95% CI outcome measures (adjusted for baseline using ANCOVA) at each assessment time point.

Outcome	2-week follow-up		4-week follow-up		6-week follow-up	
	(Mean ± SD)		(Mean ± SD)		(Mean ± SD)	
	CTTM	Control	CTTM	Control	CTTM	Control
	(Mean ± SD)	(Mean ± SD)	(Mean ± SD)	(Mean ± SD)	(Mean ± SD)	(Mean ± SD)
Visual analog scale (VAS 0–10 cm)	3.73 ± 1.22	4.4 ± 1.37	2.9 ± 0.95	3.5 ± 1.27	2.60 ± 0.72	2.9 ± 1.06
Difference (95% CI)	0.90 (0.54 to 1.26)		0.79 (0.42 to 1.5)		0.44 (0.11 to 0.76)	
*P* value	<0.05		<0.05		<0.05	
Tissue hardness (%)	48.85 ± 11.29	49.80 ± 10.45	46.20 ± 7.54	49.51 ± 7.85	48.96 ± 8.01	47.41 ± 8.62
Difference (95% CI)	−2.38 (−7.42 to 2.65)		4.30 (0.70 to 7.89)		0.97 (−3.27 to 5.22)	
*P* value	0.347		<0.05		0.647	
Pressure pain threshold (kg/cm^2^)	3.72 ± 0.60	3.17 ± 0.65	4.01 ± 0.62	3.4 ± 0.68	4.12 ± 0.55	3.53 ± 0.73
Difference (95% CI)	0.35 (0.13 to 0.57)		0.32 (0.09 to 0.55)		0.38 (0.13 to 0.63)	
*P* value	<0.05		<0.05		<0.05	

*Note*. CTTM is court-type traditional Thai massage. NA is not available. *P* < 0.05 is statistically significant differences from baseline.
